# The TDR MOOC training in implementation research: evaluation of feasibility and lessons learned in Rwanda

**DOI:** 10.1186/s40814-020-00607-z

**Published:** 2020-05-15

**Authors:** Cole Hooley, Ana A. Baumann, Vincent Mutabazi, Angela Brown, Dominic Reeds, W. Todd Cade, Lisa de las Fuentes, Enola K. Proctor, Stephen Karengera, Kenneth Schecthman, Charles Goss, Pascal Launois, Victor G. Davila-Roman, Eugene Mutimura

**Affiliations:** 1 Brown School of Social Work, WUSTL, St. Louis, MO USA; 2 Regional Alliance for Sustainable Development (RASD) Rwanda, Kigali, Rwanda; 3 Cardiovascular Division, WUSTL, St. Louis, MO USA; 4 Division of Geriatrics and Nutritional Science, WUSTL, St. Louis, MO USA; 5 Program in Physical Therapy, WUSTL, St. Louis, MO USA; 6 Division of Biostatistics, WUSTL, St. Louis, MO USA; 7 Special Programme for Research and Training in Tropical Diseases, WHO, Geneva, Switzerland

**Keywords:** Training, D&I competencies, Low-middle-income countries, Hypertension

## Abstract

**Background:**

Hypertension (HTN) affects nearly 1 billion people globally and is a major cause of morbidity and mortality. In low- and middle-income countries (LMICs), HTN represents an unmet health care gap that can be addressed by strengthening national health care systems. The National Heart, Lung, and Blood Institute recently funded the T4 Translation Research Capacity Building Initiative in Low Income Countries (TREIN) program to build capacity in dissemination and implementation (D&I) research in HTN in LMICs. The Special Programme for Research and Training in Tropical Diseases (TDR) at the World Health Organization (WHO) recently developed a massive open online course (MOOC) to train in D&I. Herein, we report on the use of the TDR WHO MOOC in D&I for the TREIN program in Rwanda, assessing feasibility of the MOOC and D&I competencies after MOOC training.

**Methods:**

Participants in one-group MOOC training completed pre- and post-training questionnaires to assess dissemination and implementation (D&I) competency outcomes and feasibility. D&I competencies were measured by use of a scale developed for a US-based training program, with the change in competency scores assessed by paired *t* test. Feasibility was measured by completion of homework and final project assignment and analyzed using descriptive statistics.

**Results:**

Of the 92 trainees enrolled, 35 (38%) completed all MOOC components. D&I competency scores showed strong evidence of improvements from pre- to post-test. The full-scale average score improved by an average of 1.09 points, representing an effect size of 1.25 (CI 0.48-2.00); all four subscales also showed strong evidence of improvements. Trainees reported challenges to MOOC course completion that included technological issues (i.e., limited internet access) and competing demands (i.e., work, family).

**Conclusions:**

In the context of LMIC training, the MOOC course was feasible and course completion showed improvement in D&I competency scores. While the program was designed with a focus on training for tropical diseases, there is potential for scalability to a wider audience of health care researchers, workers, administrators, and policymakers in LMIC interested in D&I research in non-communicable diseases.

## Background

Non-communicable diseases in general and hypertension in particular are the major causes of disability and death worldwide. The World Health Organization (WHO) estimates that deaths caused by cardiovascular diseases, of which hypertension is the major contributor, are threefold higher in low- and middle-income countries (LMICs) vs. high-income countries [1–3]. The development of robust health systems is imperative to tackle the challenges associated with the epidemic of NCDs and HTN. The National Heart, Lung and Blood Institute (NHLBI) recently funded the T4 Translation Research Capacity Building Initiative in Low Income Countries (TREIN) program to build capacity in dissemination and implementation (D&I) research in non-communicable diseases and particularly in hypertension in LMICs. The Special Programme for Research and Training in Tropical Diseases (TDR) at the World Health Organization (WHO) recently developed a massive open online course (MOOC) to train in D&I research. Our team in Rwanda and at Washington University in St. Louis partnered with the TDR at the WHO to use their Massive Open Online Course (MOOC) on D&I research.

As part of its 2018–2030 Strategic Planning [4], the Special Programme for Research and Training in Tropical Diseases aims to strengthen capacity building with the goal of overcoming barriers to the implementation of health interventions. With the hypothesis that local LMIC-driven research can have high-impact, the D&I research toolkit [5] and free online introductory MOOC were developed. While the MOOC focuses on infectious diseases, many implementation science concepts are cross-disciplinary and can be easily adapted to non-communicable diseases and hypertension control. Additional benefits to our stakeholders included participation in a structured mentoring program in which peers work together to solve problems, an important component for capacity building [6].

The partnership between Washington University in St. Louis, RASD Rwanda, the Rwandan Ministry of Health, the University of Rwanda, major teaching hospitals and stakeholders, and the Special Programme for Research and Training in Tropical Diseases at WHO was formed to support capacity building in D&I research. Our team followed Potter and Brough’s [7] model of elements for systemic capacity building, which identifies a pyramid of nine separate but interdependent components in a four-tier hierarchy of capacity building needs: (1) structures, systems, and roles; (2) staff and facilities; (3) skills; and (4) tools [7]. Accordingly, the partnership has sought to build skills and equip stakeholders with tools. In 2018, the team implemented a week-long training on the topics of hypertension and cardiovascular diseases, dissemination and implementation science, biostatistics, and research career development. The team also visited and met with stakeholders at the University of Rwanda, major teaching hospitals, and community healthcare facilities to foster clear bi-directional partnerships [8]. The current report builds on our 2018 capacity-building work after the summer in-person meeting. The goals of this paper are to describe the 2019 MOOC-D&I training, to share preliminary feasibility and dissemination and implementation competency outcomes of the training and to offer lessons learned that can be used to enhance future D&I training in LMICs. Feasibility was operationalized by completion of the MOOC assignments.

## Methods

### WHO D&I MOOC program

The MOOC is a step-by-step online video training (with subtitles in English, French, and Spanish) that covers five modules over six weeks, including (1) identify the challenges of various health settings, (2) assess the appropriateness of existing disease control strategies, (3) develop new interventions and strategies by working with communities and stakeholders, (4) specify implementation research questions, and (5) design rigorous research projects, including identifying implementation research outcomes, evaluating effectiveness, and making plans to scale-up implementation in real-life settings. The MOOC comprises of four milestones that lead to a certificate, as follows: (1) complete homework after each session, (2) satisfactorily pass four quizzes (one for each of the first four first modules), and (3) complete a capstone project at the end of the fifth module. Mentors affiliated with the MOOC program grade the homework; the capstone project is graded by both mentors and MOOC peer trainees, and the quizzes are graded by the computer software.

### Trainees

As the MOOC is open by invitation only, in collaboration with the MOOC representative (Dr. Pascal Launois), RASD-Rwanda (local NGO) invited potential trainees to apply. MOOC trainees included healthcare workers, researchers, and faculty. Trainees were employed by the Rwandan Ministry of Health, the University of Rwanda College of Medicine and Health Sciences, district hospitals, or other health care facilities. Eligibility criteria included public health researchers and decision-makers, disease control program managers, academics, or others involved in hypertension control programs. No technical or scientific background in implementation science was needed for enrollment, although having a background in health sciences was advantageous. Of the 132 subjects invited to apply, 92 enrolled in the MOOC training.

The unit of analysis was individual trainees in the Special Programme for Research and Training in Tropical Diseases D&I research MOOC. All individual trainees were assigned to the training condition. Given that this study represents an initial pilot study and was intended to explore feasibility of the training, a sample size was not pre-determined but rather the goal was to enroll as many trainees as possible. There was no blinding in the study. The MOOC ran during October 13 through December 14, 2018. Each learning module lasted one week, but the modules remained open until the closure of the MOOC to allow delayed completion.

### Design and outcomes

This study had two outcomes: training feasibility and competency in D&I research. MOOC completion using the training’s milestone data was used to assess MOOC feasibility [9]. MOOC certificate milestones, which included the four quizzes, and the capstone project, were monitored for each trainee. To assess the competency outcome of the MOOC, a one-group, pre-post study design was used. All Rwandan MOOC trainees were invited to complete a D&I competencies scale [10–12] at the beginning of the MOOC training and again at the end. The competencies instrument was developed for other US-based D&I trainings [10, 12]. The instrument includes 43 competencies divided into four subscales: D&I research skills, theories and research approaches, study designs and analysis, and practice-based considerations [10, 12]. MOOC enrollees rated themselves on each competency using a 5-point Likert scale (1, not at all, to 5, extremely). The survey was completed via Qualtrics, an online survey platform.

To enhance our understanding of the experiences of participants with the training, after the MOOC course was completed, a member of the research team emailed all MOOC participants soliciting feedback regarding barriers to training, addressing MOOC course and internet connectivity and/or other issues. Qualitative responses from 20 participants were received; all respondents had completed at least one module and the respective quiz. The email responses from MOOC trainees were compiled and reported without further qualitative analysis.

### Statistical analysis

Univariate statistics were conducted to analyze the training feasibility outcome (i.e., homework completion), and a paired *t* test to assess change in D&I competencies between pre- and post-test. Trainee average D&I competency subscale scores and total score from their pre- and post-assessments were calculated [12]. A sensitivity analysis with *t* tests was conducted using the sample of trainees with partial responses, with full responses, and with full responses removing any outliers. Each model showed strong evidence of improvement from pre- to post-test, with the magnitude of pre-to-post differences decreasing as the sample became more conservative. The findings for the most conservative sample which included trainees with a complete pre- and post-D&I competency scale and the removal of one outlier are reported (*n* = 16). All data management and analysis was done in Stata v.14.2.

## Results

### MOOC participation

**Fig. 1:**
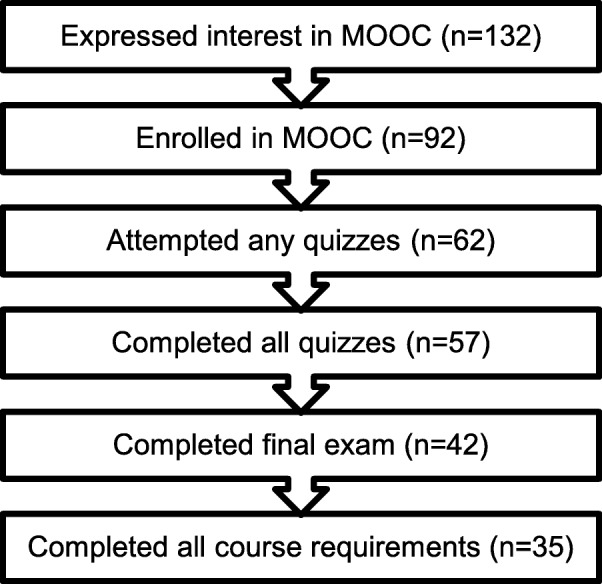
MOOC training participation attrition

Of the 132 potential trainees invited to participate in the MOOC program, 92 enrolled, 62 completed the first quiz, 58 completed the second and third, 57 completed the fourth, 42 took the final exam, and 35 (38% MOOC completion rate) completed all requirements to receive the training certificate (Fig. 1). Training completion was the primary outcome to measure training feasibility. The largest attrition occurred between enrollment and first quiz, as 33% of those who enrolled did not take the first quiz. The second largest point of attrition was between quiz 4 and the final test (26% attrition).

### D&I competency

Prior to starting the MOOC training, a pre-training survey was sent to all potential trainees, *n* = 132 (3 email addresses bounced, *n* = 129); 74 trainees responded to the pre-training survey and 43 trainees completed the post-training survey. Of the 43 trainees, 17 had complete pre- and post- D&I competency scales. One outlier was removed from the sample of trainees, reducing the sample to *n* = 16.

Table 1 shows demographic characteristics for those who completed both pre- and post-competency surveys (*n* = 43). The majority of the trainees were male (65%), the average age of trainees was 35 years old (range 25–61), and average years of experience was 4.32 (range 0–15). Over half had a master’s degrees (56%), most worked for the University of Rwanda (53%), most had not participated in an earlier D&I training held during the summer of 2018 (63%), and most did not have or were unsure if they had a D&I mentor (52%). Approximately one third of trainees described their position as assistant lecturer (30%).

**Table 1:** MOOC trainee demographics from Rwanda, overall (*n* = 43), and for those who completed the pre- and post-competency scores (*n* = 16)

	*N* = 43	*N* = 16
**Gender,*n*(%)**		
Male	28 (65%)	12 (75%)
Female	14 (33%)	4 (25%)
Missing	1 (2%)	0%
**Age (*m*± sd, range)**		
Trainee age	35 ± 7 (25–61)	35 ± 6.04 (25-46)
Years of experience	4.3 ± 3.9 (0-15)	4.2 ± 3.43 (1-13)
**Education,*n*(%)**		
PhD/MD	3 (7%)	2 (13%)
Masters	24 (56%)	9 (56%)
Some graduate	2 (5%)	1 (6%)
Bachelors	13 (30%)	4 (25%)
Missing	1 (2%)	0%
**Employer,*n*(%)**		
Ministry of Health	1 (2%)	0%
RASD Rwanda	1 (2%)	0%
Rwanda Biomedical Center	3 (7%)	1 (6%)
University of Rwanda	23 (53%)	8 (50%)
Kigali University Hospital	3 (7%)	1 (6%)
Butare University Hospital	2 (5%)	1 (6%)
District Hospital	4 (9%)	1 (6%)
Other (e.g., NGO, hospital)	5 (12%)	4 (25%)
Missing data	1 (2%)	0%
**Position,*n*(%)**		
Assistant lecturer	13 (30%)	6 (38%)
Other professional	8 (19%)	4 (25%)
Tutorial assistant	6 (14%)	2 (13%)
Physician	5 (12%)	2 (13%)
Administrator	4 (9%)	1 (6%)
Medical resident	4 (9%)	0%
Lecture	2 (5%)	1 (6%)
Missing data	1 (2%)	0%

Competency scores showed strong evidence of improvement (pre-test vs. post-test D&I scores, *n* = 16 for all, Table 2) as follows: the average full-scale score improved by 1.09 points (pre-test 2.91 ± 1.00 to post-test 4.00 ± 0.70, *t*(15) = 5.16), a change that corresponds to an effect size of 1.25 (CI 0.48–2.00). The “definitions, background, and rationale” subscale improved by 1.11 points (pre-test 3.11 ± 1.05 to post-test 4.22 ± 0.47, *t*(15) = 4.6817), corresponding to an effect size of 1.35 (CI 0.57–2.11). The “theory and approaches” subscale improved by 1.00 points (pre-test 2.99 ± 1.04 to post-test 3.99 ± 0.74, *t*(15) = 4.4173), corresponding to an effect size of 1.11 (CI 0.35–1.85). The “design and analysis subscale” scores improved by 1.05 points (pre-test 2.79 ± 1.05 to post-test 3.84 ± 0.79, *t*(15) = 4.57), corresponding to an effect size of 1.13 (CI 0.38–1.87). The final subscale, “practice-based considerations”, improved by 1.18 points (pre-test 2.83 ± 1.01 to post-test 4.01 ± 0.84, *t*(15) = 5.16), corresponding to an effect size of 1.26 (CI 0.49–2.02).

**Table 2:** D&I competencies, pre- to post-test change in average scores (scale: 1 = not at all; 5 = extremely, *n* = 16)

	Pre-test	Post-test	95% CI mean difference
Definitions, background, and rationale	3.11 ± 1.05	4.22 ± 0.47	.60–1.60
Theory and approaches	2.99 ± 1.04	3.99 ± 0.74	.52–1.48
Design and analysis	2.79 ± 1.05	3.84 ± 0.79	.56–1.54
Practice-based considerations	2.83 ± 1.01	4.01 ± 0.84	.69–1.66
Full scale	2.91 ± 1.00	4.00 ± 0.70	.64–1.53

Sensitivity analyses were conducted with different samples to ensure the robustness of our conclusions. The results for the pre/post *t* test sample included the one outlier with complete pre/post responses, in addition to the previously reported analytic sample, are as follows (*n* = 17 for all). The D&I competency scores improved from pre- to post-test: the average full-scale score improved by 1.23 points (pre-test 2.81 ± 1.07 to post-test 4.04 ± 0.69, *t*(16) = 4.9909). The “definitions, background, and rationale” subscale improved by 1.23 points (pre-test 3.01 ± 1.12 to post-test 4.24 ± 0.47). The “theory and approaches” subscale improved by 1.15 points (pre-test 2.87 ± 1.11 to post-test 4.02 ± 0.74). The “design and analysis subscale” scores improved by 1.18 points (pre-test 2.69 ± 1.10 to post-test 3.87 ± 0.78). The final subscale, “practice-based considerations”, improved by 1.33 points (pre-test 2.72 ± 1.07 to post-test 4.05 ± 0.84).

The second sensitivity test results included the analytic sample, the outlier, and the other respondents who completed the pre-/post-survey but only had partial responses on the D&I competency scale. Their partial responses allowed us to create an average score. The results of the second sensitivity test are as follows (*n* = 29 for all). The D&I competency scores improved from pre- to post-test: the average full-scale score improved by 1.37 points (pre-test 2.63 ± 1.10 to post-test 4.00 ± 0.71, *t*(28) = 6.8577). The “definitions, background, and rationale” subscale improved by 1.28 points (pre-test 2.98 ± 1.13 to post-test 4.26 ± 0.49). The “theory and approaches” subscale improved by 1.37 points (pre-test 2.60 ± 1.19 to post-test 3.97 ± 0.74). The “design and analysis subscale” scores improved by 1.36 points (pre-test 2.51 ± 1.13 to post-test 3.87 ± 0.83). The final subscale, “practice-based considerations”, improved by 1.44 points (pre-test 2.60 ± 1.12 to post-test 4.04 ± 0.82). Across all three analyses, the conclusion remains unchanged: D&I competency scores showed strong evidence of improvement for all pre- to post-test comparisons.

### Factors influencing trainee experience with the MOOC

MOOC participants were solicited feedback regarding barriers to training, addressing MOOC course and internet connectivity and/or other issues. Trainees reported numerous barriers to the MOOC course. Many trainees reported that this course represented their first experience with internet-based training and raised concerns with apprehension and unclear expectations. All trainees stated that they have a full-time job with demanding schedules and raised concerns regarding the time commitment for course completion. Many completed the course during their personal time (i.e., evenings or weekends), which posed challenges related to conflicting priorities (i.e., family time vs. work). The MOOC’s tight deadlines (i.e., completion of assignments) often posed challenges. Finally, many shared that completion of the final report was a big challenge for several reasons, the most commonly cited being that their expertise in implementation science was limited. Barriers regarding internet connectivity were raised by many trainees: high-variability of internet availability and inability to connect on a regular basis affected timely completion of assignments.

## Discussion

Hypertension care is a pressing health problem in Rwanda. While there are treatment guidelines to strengthen care, selecting, adapting, and implementing these guidelines in low-middle-income countries is challenging [13]. One of the barriers in improving the quality of care for hypertension treatment is the limited capacity and infrastructure for T4 translational research. To address this issue, our research team has been training Rwandan stakeholders in D&I to strengthen research infrastructure and human capacity [7]. Our ultimate goal is to co-create robust D&I strategies to narrow the HTN care gap in Rwanda. This study examined whether the Special Programme for Research and Training in Tropical Diseases’ MOOC could be used to provide D&I training in Rwanda.

Our results show that 38% of those who officially enrolled completed the program. Historically, MOOC completion rates have varied [14–18]. In general, completion rates have been low [14–16], with some estimates as low as 6.5% [17]. The trainee completion rates were much higher than many [14–17] and are comparable to the higher completion rates (e.g. 53%) reported by others [18]. Notwithstanding trainees expressing interest in and the utility of the training, they noted multiple barriers to complete the training’s requirements. These barriers included challenges with the internet and the tight deadlines of the course. Being able to complete assignments and technology issues are barriers others have noted in regards to MOOC completion [19, 20].

The relatively high MOOC completion rate in the study’s sample was most likely influenced by additional supports offered by the research team. For example, meetings were held with the trainees so they could meet the research team and previous MOOC trainees. During this meeting, trainees received an overview of the course, its form, modules, key dates, etc. Trainees also participated in Q&A sessions to receive support for any challenges that had been raised. The research team also provided reminders and additional training sessions to manage technology-based questions. Other studies have found that similar strategies are important to support MOOC engagement [21]. It is unclear what the attrition rate with regards to our partners would have been without these additional supports.

The results also indicate that the D&I competency scale developed for a US-based training in D&I with a focus on cancer [10, 12] has potential utility to evaluate other D&I training programs. These D&I competencies are described as self-efficacy skills and the present study’s results are similar to the outcomes of other D&I trainings showing an increase between pre- to post-test [12]. The skill that had the largest change was the “definitions, background, and rationale” subscale and the skills with the least change were the “theory and approaches” and “design and analysis” subscales, which corroborates with findings from other trainings [12]. A caveat to the present evaluation is that there were only two data points. The trainees will be invited to a second in-person training in the summer of 2019. Furthermore, the Special Programme for Research and Training in Tropical Diseases is developing D&I research competencies for LMICs. Previous data suggest that, if mentored in D&I skills, trainees show continued improvement in skills level over time [22]. It is our hope that we can continue fostering their learning and progress in advancing their D&I research skills.

### Limitations

The major limitations of this study are twofold: First, there was a high attrition rate in the MOOC training course (92 enrolled, 35 completed the course). This is attributed in great part to the barriers to training identified by the post-course survey, including apprehension and unclear expectations of the course, competing demands due to work and personal/family time, a significant time commitment and tight deadlines for course completion, and limited/inconsistent access to internet. We are working with our team to address these barriers for our next MOOC cohort (see Table 3). Second, there was a high attrition rate in survey follow-up. Of the MOOC participants, 74 responded to the pre-survey and 43 participants also responded to the post-survey (58% attrition from pre to post). Of those participants who returned both the pre- and post-survey (*n* = 43), only 17 participants completed all the D&I competency questions. One of those individuals was dropped from the analysis because they were an outlier, yielding an analysis sample with only 37% of the 43 surveys. Analysis showed that the group with missing D&I competency data did not differ demographically from those with complete D&I competency data. Two sensitivity tests were conducted to assess the durability of the findings. Both tests yielded the same conclusion that MOOC participants’ D&I competence improved. Of note, of the 35 MOOC participants who completed the training, 86% had complete pre/post D&I competency scales. It is not possible to predict how our findings may have differed if all MOOC participants would have completed the pre- and post-survey.

**Table 3:** Summary of lessons learned about feasibility

**Definition**: Feasibility was defined as completion of MOOC assignments **Main findings**: 38% (37/92) of participants completed all requirements to receive the training certificate (Fig. 1). Largest attrition occurred at two points: (a) between enrollment and first quiz and (b) between quiz 4 and the final research proposal **Lessons learned and action items for future training**: 1. Improve communication regarding course expectations 2. Increase time to submit assignments 3. Facilitate peer support by substituting single individual project for pair/group projects 4. Facilitate reliable access to internet 5. Provide video and transcript downloads to facilitate stakeholders off-line use of training materials	

Third, there was no comparator group and as such the improvement in D&I competency scores could be attributed to factors other than the course itself. Fourth, some members of the research team knew the trainees, which could result in a “Hawthorne-effect” [23], where participants may falsely over-inflate their knowledge about D&I contents after the training. Inflation of self-report assessments can be addressed with objective measures, such as scholarly outcomes (grants and publications) that examine D&I competencies [24]. As a next step, Rwandan trainees are actively participating in our ongoing research projects and/or writing grant proposals applying D&I concepts they learned. We are capturing grants submitted and awarded, as well as other scholarly outcomes (e.g., papers, conference presentations) in collaboration with our stakeholders. Finally, the completion rates were influenced by the additional supports offered by the study team. It is unclear what the completion rates would have been without those supports.

### Future directions

Notwithstanding the challenges that trainees faced, the small sample size, and the issues inherent to online training, it seems that partnering with existing infrastructure such as the Special Programme for Research and Training in Tropical Diseases MOOC is a potentially scalable, free, and high-quality strategy to train LMIC partners in D&I. Additional qualitative assessment will help identify strategies to support stakeholders in the next wave of training.

Using existing metrics, such as the D&I competencies, will allow for generalizability and further evaluation on how to design training in D&I. For example, the partnership’s next D&I training will focus on the skills that saw the least amount of change such as D&I theories and designs. Further work could be done to compare the effectiveness of different modalities (e.g., in vivo, online), dose, and frequency in D&I training across different settings in low-income countries and further identify how to objectively measure efficacy of trainings with non-academic trainees.

## Conclusions

This study described the 2019 massive open online course (MOOC) for D&I developed by the Special Programme for Research and Training in Tropical Diseases. The results show that partnering with existing infrastructures to provide D&I training for trainees in LMICs is feasible and multiple barriers need to be addressed to improve participant retention. The report shares several lessons learned to address these barriers. Additionally, the report shows that the MOOC increased self-reported D&I competencies from trainees in Rwanda. The partnership hopes that these lessons learned can help other groups in replicating and improving D&I training, particularly in LMICs.

## Data Availability

Data are available upon request
